# Talent Identification and Relative Age Effects in English Male Rugby Union Pathways: From Entry to Expertise

**DOI:** 10.3389/fspor.2021.640607

**Published:** 2021-02-19

**Authors:** Adam L. Kelly, Kevin Till, Daniel Jackson, Donald Barrell, Kate Burke, Jennifer Turnnidge

**Affiliations:** ^1^Department of Sport and Exercise, Research Centre for Life and Sport Sciences (CLaSS), School of Health Sciences, Birmingham City University, Birmingham, United Kingdom; ^2^Carnegie School of Sport, Leeds Beckett University, Leeds, United Kingdom; ^3^Rugby Football Union, London, United Kingdom; ^4^PLAYS Research Group, School of Kinesiology and Health Studies, Queen's University, Kingston, ON, Canada

**Keywords:** Rugby Football Union, age-grade rugby, talent identification, talent development, player pathway

## Abstract

A common practice in youth rugby union is to group players based on (bi)annual age with fixed cut-off dates. The overrepresentation of players born at the start of the cut-off date and the underrepresentation of players born toward the end of the cut-off date are termed relative age effects (RAEs). The aim of this study was to examine RAEs during entry into professional and international rugby union pathways in England, as well as comparing them to their respective senior cohort: U15 Regional Academy Player (*n* = 1,114) vs. Senior Professional Player (*n* = 281) and U16–23 England Academy Player (*n* = 849) vs. Senior International Player (*n* = 48). Chi-square (χ^2^) analysis compared birth quarter (BQ) distributions against expected distributions. Odds ratios and 95% confidence intervals compared the likelihood of a BQ being selected. Findings revealed a significant overrepresentation of relatively older players compared with their relatively younger peers within both youth cohorts (*P* < 0.001; BQ1 = 42.5% vs. BQ4 = 9.6%; BQ1 = 36.5% vs. BQ4 = 15.2%). In comparison, there was no significant difference in the BQ distributions within both senior cohorts. Further, BQ4s were 3.86 and 3.9 times more likely to achieve senior professional and international levels than BQ1s and BQ2s, respectively. It is suggested that relatively younger players may have a greater likelihood of achieving expertise following entry into a rugby union talent pathway due to benefitting from more competitive play against relatively older counterparts during their development (e.g., reversal effects; the underdog hypothesis). Moreover, possible solutions (e.g., age and anthropometric banding; playing-up and playing-down) are discussed to encourage practitioners and policy makers to create the most appropriate learning environment for every player.

## Introduction

Identifying young rugby union players with the capabilities to achieve expertise at adulthood is a contemporary challenge for professional clubs and national governing bodies (Sherwood et al., 2019). Developmental pathways are mapped by academies and organizations to prepare youth players for the demands of professional and international rugby union (Parsonage et al., 2014). However, the difficulty of accurately predicting future performance abilities, coupled with the complexities of the athlete development process, can result in biases during the recruitment process into talent development pathways (Baker et al., 2018; Till and Baker, 2020). One such selection bias that has been consistently highlighted in the literature is relative age effects (RAEs; Barnsley et al., 1985; Smith et al., 2018). RAEs illustrate that when athletes are banded according to (bi)annual-age groups, those who are born near the beginning of the selection year (e.g., September 1st in the United Kingdom) are often overrepresented compared with those who are born toward the end (e.g., August 31st; Cobley et al., 2009). Possible explanations that have been offered for this effect include the enhanced physiological and cognitive maturity of relatively older athletes, which allows them to outperform their younger age-matched peers (Doncaster et al., 2020). Further, Hancock et al. (2013a) proposed the social agents model to illustrate how RAEs are constructed in sport. This model suggests that social agents (e.g., parent, coaches, athletes) interpret factors, such as maturity, size, and skill, through their societal interactions, which in turn influence the quality of athletes' sport experiences. For instance, due to the “Matthew effect” (i.e., the rich get richer; Merton, 1968), parents are providing coaches with a pool of talented athletes, which is overloaded with relatively older athletes. Thus, coach selections related to relative age would be influenced by parental enrolment decisions (Hancock et al., 2013a).

Previous research suggests that the contact and invasive nature of rugby union may exacerbate the physiological advantages of those athletes who are chronologically older (Baker et al., 2009). Specifically, the rules and regulations of rugby union (e.g., tackling, running with the ball, ruck, scrum) are characterized by a broad range of physical factors, such as body size, speed, change of direction speed, high-intensity running ability, muscular strength, and power—all of which are crucial for player development (Till et al., 2020b). Thus, from an athlete development viewpoint, those born in birth quarter one (BQ1) of an annual-age group in England (i.e., September, October, November) may have developed more physically, cognitively, and socially than their later born BQ4 peers (i.e., June, July, August; Rubajczyk and Rokita, 2020). Due to the physical nature of rugby union, coaches may misconstrue older relative age, enhanced growth and maturation, and advanced fitness capacities at youth level as indicators for *performance* and *potential* (Hancock et al., 2013b; Furley and Memmert, 2016).

Skewed birthdate distributions among youth players favoring those born near the start of the cut-off date have been previously documented in rugby union (Musch and Grondin, 2001). From a recreational perspective, Lewis et al. (2015) found consistent RAEs across all age-grade and district cohorts from Under (U)7 to U19 (e.g., BQ1 = 29% vs. BQ4 = 22%). They also revealed an increasingly pronounced effect at U16 representative levels where regional and national selection occurs (e.g., BQ1 = 44% vs. BQ4 = 12%). Likewise, Roberts and Fairclough (2012) examined the North West of England representative squads from U13 to U16, illustrating a significant overrepresentation of those born in BQ1 (46%) compared with those born in BQ4 (14%). From an English Premiership rugby union academy outlook, McCarthy and Collins (2014) identified a significant overrepresentation of BQ1s (48%) compared with BQ4s (8%) in a single club. Collectively, these results suggest that the higher performance status of the youth level cohort, the more skewed the BQ distribution becomes.

RAEs in male rugby union have also been identified in other popular countries, such as Australia (Fernley, 2012), France (Delorme et al., 2009), New Zealand (Simons and Adams, 2017), and South Africa (Grobler et al., 2016). While exploring whether RAEs existed at the senior international level, Kearney (2017b) adopted a cross-cultural comparison as part of his methodology. In contrast to the youth case studies, he illustrated that only South Africa had pronounced RAEs across all playing positions at the senior level, suggesting that differences in national sport culture may be an important consideration while exploring who is at risk of RAEs. This also suggests that RAEs are considerably less prominent at senior levels than at youth levels in rugby union. Further, although these studies offer further evidence of RAEs, it is difficult for them to provide a true reflection of the England professional and international organizational structures on a national scale.

When exploring RAEs during the transition from academy to professional level at an English Rugby Premiership club, McCarthy and Collins (2014); McCarthy et al. (2016) identified a *reversal effect* of relative age. Specifically, they found that despite RAEs at the academy level favoring relatively older players (e.g., BQ1 = 48% vs. BQ4 = 8%), there was a greater proportion of relatively younger players who successfully converted to professional level (e.g., BQ1 = 20% vs. BQ4 = 50%). Furthermore, from a senior international level perspective, Jones et al. (2018) found that BQ1s were significantly underrepresented in 12 out of 14 criteria based on international caps and performance, whereas BQ4s were overrepresented in 8 out of 14 criteria (although this was not statistically significant). Jones et al. (2018) proposed that these late birthday benefits may be due to *survival of the fittest concept*s (e.g., Collins and MacNamara, 2012). Similar outcomes are apparent in other sports, such as rugby league (Till et al., 2016) and football (Kelly et al., 2020b), referred to as the *underdog hypothesis* by Gibbs et al. (2012). These studies illustrate the importance of combining both youth and senior representatives together to better understand who is at risk of RAEs, as well as identifying the potential mechanisms of the youth to senior level transitions. Furthermore, these studies also emphasize the need to identify RAEs along developmental trajectories to offer a greater longitudinal perspective. Indeed, the necessity of examining RAEs at more than one point in time is something that was recently encouraged by Schorer et al. (2020).

In this current study, the authors explored two English rugby union player pathways: (a) professional pathway—England Rugby Premiership (i.e., U15 Regional Academy Player to Senior Professional Player) and (b) international pathway—England Rugby Football Union (i.e., U16–U23 England Academy Player to Senior International Player). During the first pathway, academy programs are delivered *via* 14 Regional Academies (aligned with 12 England Rugby Premiership clubs, 1 England Rugby Championship club, and 1 unaffiliated). Individuals are typically identified from community or school rugby union and are selected to be a *U15 Regional Academy Player* from aged 14 years prior to potentially signing as a *Senior Professional Player* at aged 18 years (Till et al., 2020b). During the second pathway, certain individuals from the regional academy age groups who have displayed the potential to become a future senior professional or *Senior International Player* are selected to be a *U16–U23 England Academy Player*. The aim of this study was to explore the BQ distributions of the four cohorts within these two player pathways in England. Moreover, to examine the likelihood of achieving senior professional and international status, the Senior Professional Player cohort was compared against the U15 Regional Academy Player BQ distribution, whereas the Senior International Player cohort was compared against the U16–U23 England Academy Player BQ distribution.

## Methods

### Sample and Procedure

Each player was allocated into one of the four cohorts based on their playing level: (a) U15 Regional Academy Player (*n* = 1,114), (b) Senior Professional Player (*n* = 281), (c) U16–U23 England Academy Player (*n* = 849), and (d) Senior International Player (*n* = 48). Every registered player during the last three seasons (i.e., 2016/17, 2017/18, 2018/19^1^) within these four cohorts participated in this study (total *n* = 2,292). In accordance with English annual-age group cut-off dates, this methodology divided the year into four 3-month BQs, starting with September 1st as *month 1* and ending with August 31st as *month 12*^2^ (e.g., Till et al., 2009). Thus, each player was assigned a BQ corresponding to their birthdate to create an observed BQ distribution within each of the four cohorts. The observed BQ distributions from each cohort were subsequently compared against the expected BQ distribution calculated from average national live births (i.e., National Norms applied from the Office for National Statistics, 2015; see Delorme and Champely, 2015 for a review). To examine the likelihood of achieving senior professional and international status, further comparisons were provided for the two respective player pathways: (a) professional pathway and (b) international pathway. As such, the Senior Professional Player cohort was compared against the expected U15 Regional Academy Player BQ distribution, whereas the Senior International Player cohort was compared against the expected U16–U23 England Academy Player BQ distribution, respectively. The study was ethically approved at both organizational (England RFU) and institutional (Birmingham City University) levels.

### Data Analysis

A chi-square (χ^2^) goodness of fit test was used to compare the BQ distributions of each cohort against the expected BQ distributions, following the procedures outlined by McHugh (2013). Since this test does not reveal the magnitude of difference between the BQ distributions for significant χ^2^ outputs, Cramer's V was also used. The Cramer's V was interpreted as per conventional thresholds for correlation, whereby a value of 0.06 or more indicated a small effect size, 0.17 or more indicated a medium effect size, and 0.29 or more indicated a large effect size (Cohen, 1988). Odds ratios (ORs) and 95% confidence intervals (CIs) were calculated in order to compare the likelihood of each BQ being represented (CIs including 1 marked no association). Results were considered significant for *P* < 0.05.

## Results

There was a significant difference between the BQ distributions of the U15 Regional Academy Player cohort compared with the National Norms [χ^2^ (df = 3) = 252.880, *P* < 0.001, V = 0.34]. The ORs showed an increased likelihood of relatively older players being selected, with the highest OR being BQ1 vs. BQ4 (4.23; 95% CI 3.38–5.77). Similarly, there was a significant difference between the BQ distributions of the U16–U23 England Academy Player cohort compared with the National Norms [χ^2^ (df = 3) = 83.172, *P* < 0.001, V = 0.22]. The ORs showed an increased likelihood of relatively older players being selected, with the highest OR being BQ1 vs. BQ4 (2.40; 95% CI 1.82–3.17). However, there were no significant differences between the BQ distributions of both the Senior Professional Player and Senior International Player cohorts when compared with the National Norms (see Table 1).

**Table 1:** The BQ distributions of the professional and international pathways.

**Cohort** **(National norms)**	**BQ1** **(25.46%)**	**BQ2** **(24.47%)**	**BQ3** **(24.65%)**	**BQ4** **(25.42%)**	**Total**	**χ2 (df = 3)**	***P***	**Cramer's V**
U15 Regional Academy Player	474 (283.62)	311 (272.60)	222 (274.60)	107 (283.18)	1114	252.880	<0.001	0.34
U16-U21 England Academy Player	310 (216.16)	232 (207.75)	178 (209.28)	129 (215.82)	849	83.172	<0.001	0.22
Senior Professional Player	77 (71.54)	64 (68.76)	73 (69.27)	67 (71.43)	281	1.222	0.748	0.05
Senior International Player	12 (12.22)	6 (11.75)	17 (11.83)	13 (12.20)	48	5.124	0.163	0.23

**Figure 1:**
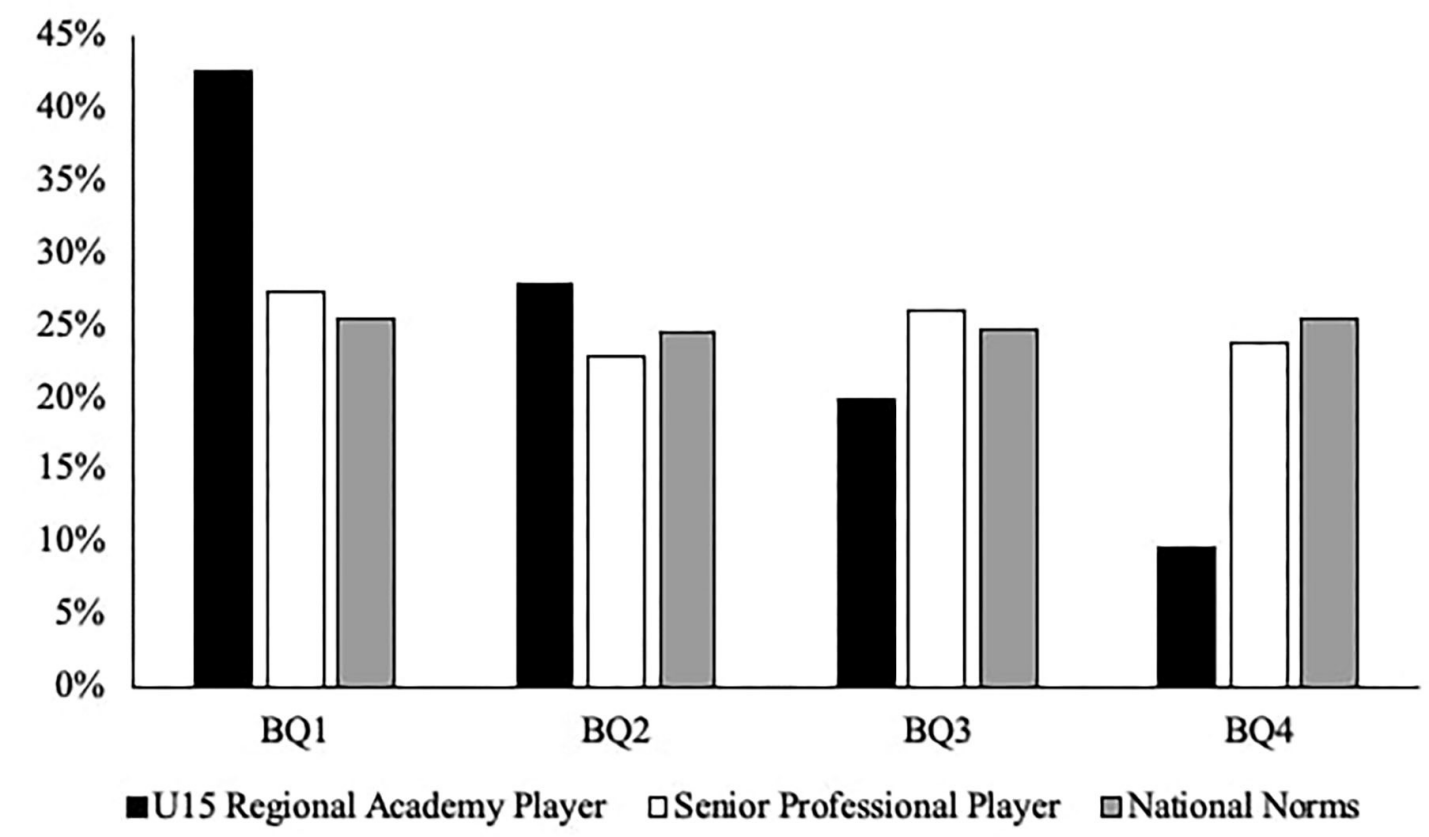
The BQ distribution percentages of the professional pathway.

**Figure 2:**
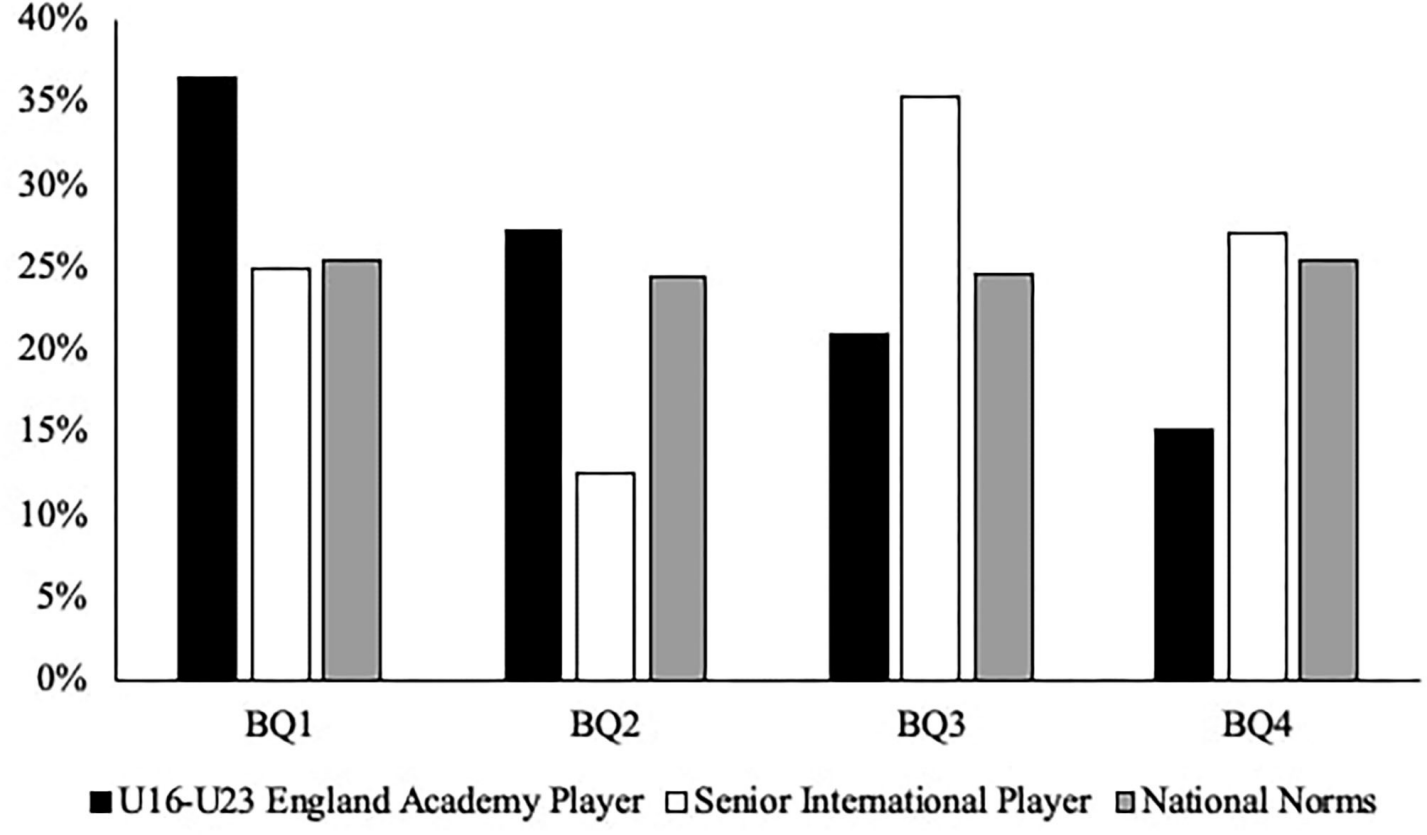
The BQ distribution percentages of the international pathway.

When analyzing the respective pathways, there was a significant difference between the BQ distributions of the Senior Professional Player cohort compared with the expected U15 Regional Academy Player cohort [χ^2^ (df = 3) = 82.358, *P* < 0.001, V = 0.38; see Figure 1]. When comparing the youth and senior distributions, the ORs showed an increased likelihood of relatively younger players achieving professional status, with the highest OR being BQ4 vs. BQ1 (3.86; 95% CI 2.27–6.56). Likewise, there was a significant difference between the BQ distributions of the Senior International Player cohort compared with the expected U16–U23 England Academy Player cohort [χ^2^ (df = 3) = 14.851, *P* = 0.002, V = 0.39; see Figure 2]. When comparing the youth and senior distributions, the ORs showed an increased likelihood of relatively younger players achieving international status, with the highest OR being BQ4 vs. BQ2 (3.90; 95% CI 1.04–12.89).

## Discussion

The purpose of this study was to explore the BQ distributions of two youth (i.e., U15 Regional Academy Player and U16–U23 England Academy Player) and two senior (i.e., Senior Professional Player and Senior International Player) rugby union cohorts. Findings revealed that there was a significant overrepresentation of players born earlier in the selection year compared with their later born peers within both of the youth cohorts (e.g., U15 Regional Academy Player—BQ1 = 42.5% vs. BQ4 = 9.6%; U16–U23 England Academy Player—BQ1 = 36.5% vs. BQ4 = 15.2%). However, there was no significant difference in the BQ distributions within both of the senior cohorts (e.g., Senior Professional Player—BQ1 = 27.4% vs. BQ4 = 23.8%; Senior International Player—BQ1 = 25% vs. BQ4 = 27.1%). Despite no RAEs prevalent at the senior levels, the skewed BQ distributions within both of the youth cohorts highlight that RAE differences exist along the professional and international rugby union pathways in England.

To examine the likelihood of achieving professional and international status based on existing distributions from youth and senior levels, further analysis compared the BQ distributions of the senior cohorts against the expected BQ distribution from their respective youth cohort. In the professional pathway, findings revealed that BQ4s were 3.86 times more likely to achieve professional status at senior level than BQ1s. Similarly, in the international pathway, findings revealed that BQ4s were 3.9 times more likely to achieve international status at senior level than BQ2s. Based on existing BQ distributions, this demonstrates that relatively later born players who are selected at youth level may be significantly more likely to achieve expertise at senior levels than their relatively earlier born peers.

This study adds a broader professional and international pathway lens to the existing literature in rugby union that explores RAEs. Indeed, these results resonate with previous youth rugby union research. Specifically, other studies in the United Kingdom have documented similar skewed BQ distributions at age-grade, regional, and academy youth levels, such as Lewis et al. (2015) (BQ1 = 29% vs. BQ4 = 23%), Roberts and Fairclough (2012) (BQ1 = 46% vs. BQ4 = 14%), and McCarthy and Collins (2014) (BQ1 = 48% vs. BQ4 = 8%), respectively. When exploring the playing level of these youth cohorts together, it appears that higher competition (e.g., regional, academy) may increase RAEs when compared with recreational level (e.g., age grade). This may be due to the competitive nature of high-performance rugby union, whereby athletes are selected based on current performance capabilities, rather than their ability to achieve expertise at senior level (Müller et al., 2015). Smith et al. (2018) have previously documented this trend in female sport during their systematic review, whereby they showed that significant OR estimates increased with playing level prior to senior competition. Thus, coaches and practitioners are encouraged to consider the long-term development outcomes of youth players when selecting at academy level, as opposed to solely focusing on existing performance abilities (Kelly and Williams, 2020). However, RAEs appeared to be more prevalent at the entry point of selection in the professional pathway at U15s than in the youth international pathway at U16–23s. Thus, key stakeholders who are recruiting players at the entry stage into academies should act with added caution.

This trend appears to continue into adulthood, whereby RAEs seem to be much less established (although less studied) at senior levels than at youth levels (e.g., Lemez et al., 2016; Kearney, 2017a,b). As such, selection at youth levels should logically turn to recruiting equal BQ distributions to reflect the opportunities presented at adulthood (Bennett et al., 2019). Although this current study does not identify players who have made the successful transition from youth to senior levels, it does identify their likelihood of transitioning based on the existing BQ distributions. Specifically, it was revealed that BQ4s have a greater likelihood of successfully transitioning from youth level to senior status than BQ1s. Moreover, the particularly lower proportion of BQ2s at senior international level also requires further inquiry. Thus, it is important to explore the potential mechanisms that facilitate such trends.

As an example, McCarthy and Collins (2014); McCarthy et al. (2016) suggest that a greater proportion of relatively younger players making the successful youth to senior transition may be due to a *reversal effect* of relative age. This is a psychologically based explanation of greater “growth” that relatively younger players experience, whereby they are initially disadvantaged during their development due to additional challenges they face (also see Jones et al., 2018). In addition, Gibbs et al. (2012) put forward the *underdog hypothesis* to highlight how relatively younger players are thought to benefit by more competitive play against relatively older counterparts throughout their development. Indeed, this may also be why there are a lower total number of senior international players born in the first half of the year (particularly in BQ2) than in the second half of the year. As such, it is important to consider how to create a “BQ4 effect” for those who may require such challenges during their development (Kelly et al., 2020b). Thus, every player should be exposed to the most appropriate settings to ensure that optimal long-term development outcomes are achieved, which can vary considerably between each individual (Abbott and Collins, 2004; Côté et al., 2014).

It is also important to note the considerably higher proportion of relatively older players (i.e., BQ1 or BQ2) who are being deselected or dropping out following the initial selection. Although relatively older players appear to be more likely to be recruited into talent development pathways (e.g., U15 Regional Academy Player, U16–U23 England Academy Player), it also seems that they comprise a greater quantity of players who are unsuccessful in achieving senior professional or international status. Thus, although relatively younger players have been reported to drop out of youth sport at young ages due to RAEs (e.g., Delorme et al., 2011), it may be suggested that this is being replicated by relatively older players at the latter stages of development during the youth to senior level transition. Moreover, Wattie et al. (2007) found that relatively older high-level ice hockey players were at increased risk of injury compared with their relatively younger peers. Due to the physicality of rugby union, it could be proposed that a similar instance of injury risk in relatively older players may suggest there is greater dropout at these ages, although further research is required to substantiate these claims. Thus, chronological age grouping may be providing a disservice to both relatively younger *and* relatively older players, although at different stages of the athlete development process. Overall, in light of these findings, it is important to consider solutions for RAEs and alternative group banding strategies to support relatively younger and relatively older players in youth rugby union.

### Potential Relative Age Solutions

There is a small, but growing, body of literature that has attempted to develop solutions for RAEs (e.g., Cobley et al., 2019; Kelly et al., 2020a). Within annual-age groups for instance, Mann and van Ginneken (2017) designed an *age-ordered shirt numbering system* to reduce RAEs in youth soccer, revealing that supporting coaches and practitioners with the knowledge that the numbers on the playing shirts corresponded with the relative age of each athlete eliminated age bias. Bennett et al. (2019) recommended a *selection quota* to moderate RAEs, whereby governing bodies regulate their participating clubs to select a minimum number of players from each BQ. Tribolet et al. (2019) suggested *avoiding early deselection*, to allow continued exposure to practice, competition, and resources without the option of being released. Kelly et al. (2020b) proposed a *flexible chronological approach*, by offering early birth quartiles (i.e., BQ1s) and late birth quartiles (i.e., BQ4s) the opportunity to “play-up” and “play-down” annual-age groups, respectively. However, these suggestions are yet to be empirically evaluated in a rugby union setting. As such, future research should explore the practical implications of these strategies as solutions to moderate RAEs specifically within a youth rugby union context.

Research on alternative grouping strategies to moderate RAEs has been sparse compared with the large body of literature exemplifying its existence. In particular, where proposed grouping solutions have been suggested, little evidence has reported their effectiveness or tested attempts to directly implement them (Webdale et al., 2019). As an example, *proficiency level-based competition* in youth sport, such as the belts used in martial arts, may be a useful approach to moderate age-related performance advantages (Chiodo et al., 2011). By adopting such approach, athletes of the same chronological age will participate in regional, national, and international competitions that are balanced due to competing against those with the same “belt” or “color” (Chiodo et al., 2011). In taekwondo for instance, since athletes begin to compete at aged 10 years, youth competitions are based on a progression to meet the physiological characteristics of the children, to preserve them from an excessive physiological strain, as well as facilitate the development of their technical and tactical skills (Casolino et al., 2012). For this reason, Casolino et al. (2012) suggest how the Italian Taekwondo Federation establishes the proficiency level of youth athletes, through taking into account their training experience (i.e., from 1 to 3 years) and technical capabilities (i.e., belt or color). However, adopting such approach into team sports (e.g., rugby union) and its usefulness in moderating RAEs is yet to be examined; thus, further exploration is warranted.

One particularly interesting strategy may be utilized from the organizational practices adopted in youth American football. For instance, in contrast to many other team sports, previous research has identified no RAEs in American football, which may lend credibility to the *age and anthropometric* bandings that are often employed to group their young players (e.g., MacDonald et al., 2009; Jones et al., 2019). As such, the physical and collision-like nature of the two sports could suggest that American football regulations may provide a useful comparison in an endeavor to moderate RAEs in youth rugby union. As such, researchers are encouraged to further explore the impact of age and anthropometric banding and its impact on RAEs.

Another recent grouping approach that has produced promising results in reducing maturity-related differences in youth sport (particularly within soccer) is *bio-banding* (see Malina et al., 2019). Bio-banding groups athletes based on maturational indicators as well as anthropometric measures, which take into account individual variability in physical characteristics during early development (Cumming et al., 2017). For instance, during their maturity-matched soccer tournament, Bradley et al. (2019) revealed that later maturing players perceived the bio-banded format afforded more opportunities to express themselves, adopt positions of leadership, and have a greater influence on gameplay. Likewise, their early maturing but same-aged peers perceived the bio-banded games as more physically and technically challenging. In addition, preliminary studies have revealed that bio-banded competition may positively adapt the outcome of skill behaviors compared with annual-age group competition (e.g., Thomas et al., 2017; Abbott et al., 2019).

Together, these grouping strategies appear to logically address one of the key mechanisms of RAEs, whereby relatively older athletes are more likely to have an advanced maturity status (Webdale et al., 2019). Moreover, since growth and maturation status are positively related to physical capacities that are particularly important to youth rugby union (e.g., speed, muscular strength, power), anthropometric and biological bandings may further reduce the potential of physiological biases that are pronounced within chronological age groups (Webdale et al., 2019; Till et al., 2020b). However, Campbell et al. (2018) revealed that New Zealand youth rugby players were 46% more likely to drop out when they were bio-banded. They suggested that an increase in the aggressive style of play and the inability to participate with age-matched peers potentially contribute to dropout. These findings reinforce that in order for bio-banding to facilitate positive developmental experiences, the rugby union environment must be manipulated to harmonize with the young rugby players' needs.

It is also important to consider how bio-banding may look in youth rugby union contexts. Indeed, current studies have primarily focused their attention on academy soccer; thus, it is difficult to understand how it would be implemented within a rugby union setting, which comprises diverse talent development pathways (Till et al., 2020a). Overall, although these group banding approaches remain untested in their value for resolving RAEs, an introduction to grouping players by height, weight, and/or some maturational variables, alongside annual-age categories, may prove fruitful in mitigating RAEs in youth rugby union. As such, further research exploring alternative group banding strategies is warranted.

### Limitations and Future Directions

The small sample size and inclusion of 3 years of retrospective data may be considered as potential limitations to this study. However, the authors worked collaboratively with an exhaustive database from the youth cohorts, while findings offer an accurate representation of the English professional and international pathways on a national scale. The current study also considered all players as one homogeneous group as position-specific data were not available. Since previous research exploring RAEs in senior rugby union has revealed inconsistent findings between playing positions (e.g., forwards vs. backs; Jones et al., 2018), further enquiry within a youth context is warranted. Moreover, while exploring the existing literature and considering the current study sample, female participants appear to be underrepresented across the discipline; thus, similar research within a female context is encouraged.

Future research should also explore the potential strategies to moderate RAEs in youth rugby union. For instance, annual-age group methods (e.g., age-ordered shirt numbering, selection quotas, avoiding early deselection, flexible chronological approach) and alternative group banding policies (e.g., age and anthropometric bands, bio-banding) could offer useful guidelines for organizations to adopt practical solutions. Further studies could also examine the underlying mechanisms of selection and progression throughout professional and international rugby union pathways, through qualitatively exploring key stakeholders' (e.g., players, coaches, practitioners) perceptions and experiences throughout the development process. As such, this may advance existing knowledge of the talent identification and development processes in rugby union (e.g., reversal effects, survival of the fittest concepts, underdog hypothesis).

## Conclusion

There appears to be a complicated relationship between entry at youth levels and achieving expertise at senior levels in rugby union. The current findings reveal that, although there are no apparent RAEs throughout the senior cohorts, there are pronounced RAEs throughout the youth cohorts. This suggests that coaches and practitioners are selecting higher numbers of relatively older players at youth level, creating a bias based on older age and greater performance capabilities. Thus, those working in youth rugby union are encouraged to consider the long-term development outcomes of players when selecting at academy level, rather than focusing on existing abilities.

While exploring the likelihood of achieving senior professional and international status, there appears to be a greater chance of relatively younger players initially identified achieving professional or international level at adulthood. Therefore, it is important to consider *why* this may occur, as well as *how* to adapt existing structures to create the most appropriate learning environment for every player to achieve their full potential. As such, possible causes and solutions were presented to offer coaches and practitioners additional annual-age group strategies and alternative group bandings to mitigate RAEs in youth rugby union. However, there appears to be a paucity in research when practically implementing and empirically evaluating solutions for RAEs in rugby union. Thus, further collaboration between key stakeholders (e.g., players, coaches, practitioners) and researchers is required to explore the potential relative age strategies to facilitate greater positive youth development outcomes, as well as qualitatively examining the performance, participation, and personal development experiences of these individuals.

## Data Availability Statement

The raw data supporting the conclusions of this article will be made available by the authors, without undue reservation.

## Ethics Statement

The studies involving human participants were reviewed and approved by Birmingham City University. Written informed consent from the participants' legal guardian/next of kin was not required to participate in this study in accordance with the national legislation and the institutional requirements.

## Author Contributions

DJ, DB, and KB primarily focused on the Methods and Results sections, whereas AK, KT, and JT contributed more to the Introduction, Discussion, and Conclusion. All authors were involved with compiling the data, as well as writing the full manuscript.

## Conflict of Interest

The authors declare that the research was conducted in the absence of any commercial or financial relationships that could be construed as a potential conflict of interest.

## Footnotes

^1^The three seasons applied to this study (i.e., 2016/17, 2017/18, 2018/19) include all the available data collected by the England Rugby Football Union for the U15 Regional Academy Player and U16–U23 England Academy Player cohorts. Thus, the last three season's data from the senior cohorts (i.e., Senior Professional Player and Senior International Player) were logically matched as a comparison.

^2^Non-Northern Hemisphere players' BQs were adjusted to reflect their rugby union affiliation cut-off dates with January 1^st^ as *month 1* and ending with December 31^st^ as *month 12*.
